# Comparative evaluation of metagenomic and targeted next-generation sequencing for pathogen detection in bronchoalveolar lavage fluid from patients with lower respiratory tract infections: a retrospective cohort study

**DOI:** 10.3389/fcimb.2026.1877320

**Published:** 2026-10-14

**Authors:** Rui Zhao, Heya Na, Wei Miao, Lei Liu, Jing Zhang, Yongshuai Miao, Ying Zhao, Xiaohua Li

**Affiliations:** 1 Department of Respiratory and Critical Care Medicine, Chifeng Municipal Hospital, Chifeng, Inner Mongolia, China; 2 Affiliated Chifeng Clinical Medical College of Inner Mongolia Medical University, Chifeng, Inner Mongolia, China; 3 Department of Endocrinology, Chifeng Municipal Hospital, Chifeng, Inner Mongolia, China

**Keywords:** antimicrobial stewardship, bronchoalveolar lavage fluid, lower respiratory tract infections, Metagenomic next-generation sequencing (mNGS), precision medicine, targeted next-generation sequencing (TNGS)

## Abstract

**Background:**

Rapid and precise pathogen identification is crucial for managing lower respiratory tract infections (LRTIs). While metagenomic next-generation sequencing (mNGS) and targeted next-generation sequencing (tNGS) are emerging as valuable diagnostic tools, large-scale studies directly evaluating their real-world impact on antimicrobial stewardship remain scarce.

**Methods:**

We conducted a retrospective cohort study encompassing 941 patients with suspected LRTIs who underwent bronchoalveolar lavage fluid (BALF) sequencing. Patients were evaluated in either an mNGS cohort (n = 479; DNA-only sequencing) or a tNGS cohort (n = 462; DNA and RNA sequencing). Diagnostic performance, pathogen spectrum, and clinical utility were compared against a composite final clinical diagnosis, which served as the reference standard.

**Results:**

Both sequencing platforms demonstrated diagnostic efficacy superior to traditional laboratory tests. In the tNGS cohort, polymicrobial infections were highly prevalent (93.5%). Furthermore, tNGS demonstrated high sensitivity for fastidious pathogens, identifying 54 cases of Mycobacterium tuberculosis (a cohort in which routine smears missed 77.8% of cases) and effectively detecting Pneumocystis jirovecii. The overall diagnostic sensitivity was 90.6% (95% CI: 87.5-93.1%) for mNGS and 73.4% (95% CI:69.2%-77.3%) for tNGS. Guided by tNGS results, clinicians escalated antimicrobial therapy in 22.6% of positive cases. The clinical improvement rate following this targeted escalation was 66.2%, comparable to the 66.7% improvement rate observed in patients receiving appropriate initial empirical therapy (P = 0.946).

**Conclusion:**

Both tNGS and mNGS offer substantial diagnostic value for LRTIs, with tNGS showing particular utility in identifying fastidious and low-titer pathogens. Sequencing-guided diagnostics frequently informed antimicrobial adjustments and were associated with favorable clinical progress; however, future prospective paired-testing studies are required to isolate the direct impact of sequencing on patient survival outcomes.

## 1 Introduction

Lower respiratory tract infections (LRTIs) remain a leading cause of global morbidity and mortality (GBD 2019 Antimicrobial Resistance Collaborators, 2022; Metlay et al., 2019). Rapid and precise pathogen identification is essential for guiding targeted antimicrobial therapy and improving patient outcomes. However, conventional microbiological tests (CMTs), such as culture and smear microscopy, are limited by low sensitivity and protracted turnaround times. Consequently, these traditional modalities frequently fail to meet the urgent diagnostic demands of acute and severe infections (Miller et al., 2022).

In recent years, metagenomic next-generation sequencing (mNGS) and targeted next-generation sequencing (tNGS) have emerged as pivotal tools in clinical microbiology (Gu et al., 2021). mNGS provides the distinct advantage of unbiased, pan-pathogen detection (Gu et al., 2021; Huang et al., 2023; Liang et al., 2022). Nevertheless, its routine implementation in clinical settings is hindered by prohibitive costs, complex bioinformatic requirements, and significant interference from the human host DNA background in respiratory samples, which can obscure sparse microbial signals (Peng et al., 2021; Gaston et al., 2022). Conversely, tNGS employs multiplex PCR enrichment to amplify specific pathogen targets. This targeted methodology not only reduces testing costs but theoretically mitigates host DNA interference, thereby enhancing the detection threshold for low-titer and fastidious pathogens (Diao et al., 2022; Zhu et al., 2023; Liu et al., 2024).

While both platforms exhibit substantial clinical potential, large-scale, real-world studies directly comparing their utility in distinct patient cohorts remain limited. Furthermore, empirical evidence delineating their direct impact on clinical decision-making—specifically concerning antimicrobial stewardship and ultimate patient outcomes—is urgently needed (Chen et al., 2024; Yin et al., 2024). We hypothesized that both NGS platforms would yield superior diagnostic accuracy compared to CMTs and significantly inform clinical management. To address current literature gaps, this retrospective cohort study comparatively evaluates the diagnostic performance, pathogen spectrum, and clinical utility of mNGS and tNGS using BALF samples from a robust cohort of 941 patients with suspected LRTIs.

## 2 Materials and methods

### 2.1 Study design and participants

A retrospective cohort study was conducted at Chifeng Municipal Hospital between January 2021 and December 2023. A total of 941 patients presenting with suspected lower respiratory tract infections (LRTIs) were included in the final analysis. The detailed patient screening, exclusion criteria, and enrollment workflow are illustrated in **Figure 1**. All enrolled patients underwent flexible bronchoscopy to obtain bronchoalveolar lavage fluid (BALF) samples for high-throughput sequencing. The study population was evaluated in two distinct, non-randomized cohorts: the mNGS cohort (n = 479) and the tNGS cohort (n = 462). Patient allocation to a specific sequencing platform was not randomized or blinded; rather, test selection was determined by the attending physicians based on clinical judgment, temporal availability of the assays, and patient financial considerations. To evaluate the diagnostic performance of both sequencing platforms in a real-world clinical setting, the final clinical diagnosis at discharge served as the reference standard. To minimize incorporation bias, this composite reference standard was adjudicated by a multidisciplinary diagnostic committee comprising senior pulmonologists and clinical microbiologists. The final clinical diagnosis was established through a comprehensive evaluation of presenting clinical symptoms, radiological progression, inflammatory biomarkers, conventional microbiological test results, and the patient’s clinical response to targeted therapy, rather than relying solely on sequencing outputs.

**Figure 1:**
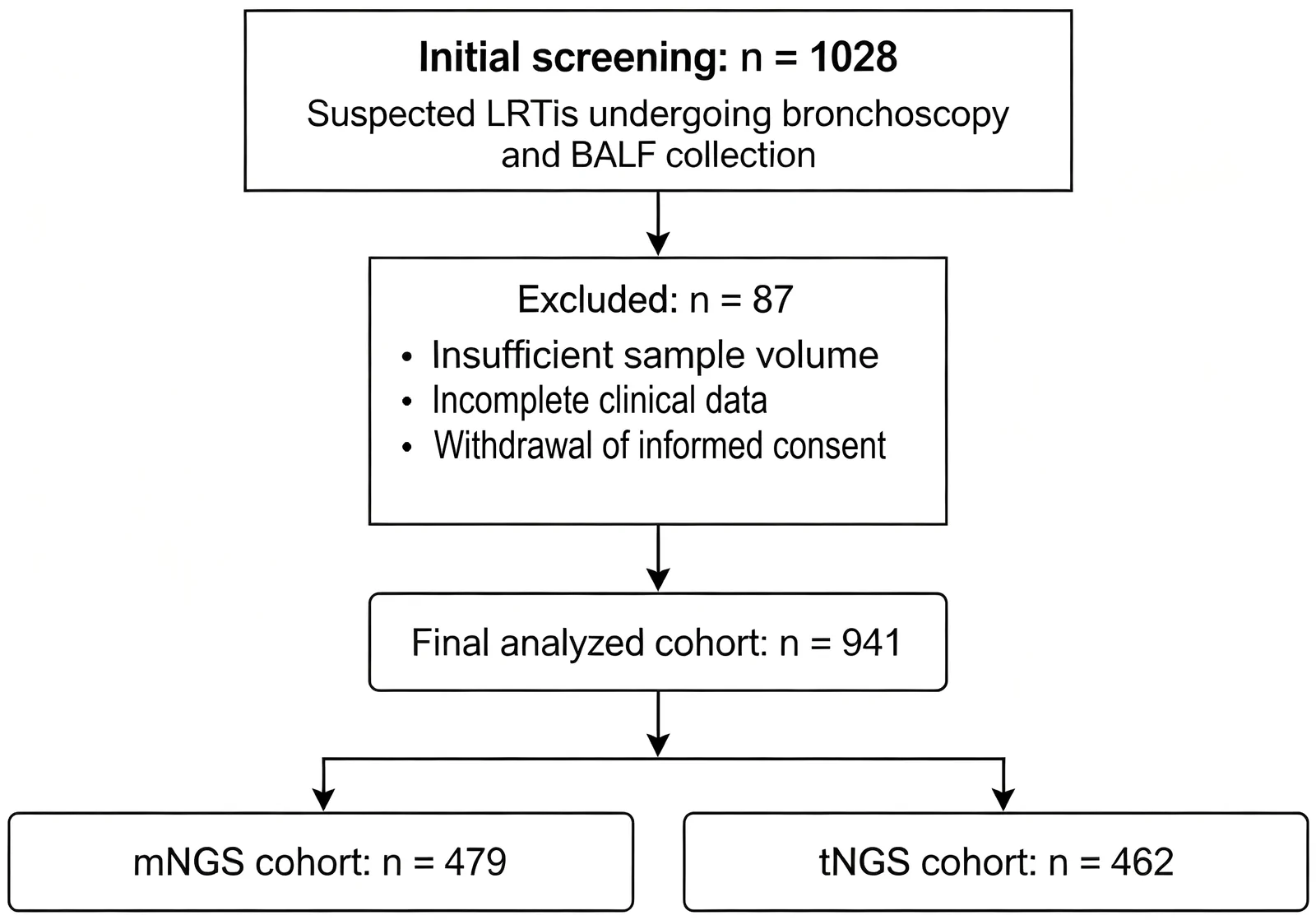
Flowchart of patient screening and enrollment. A total of 1028 patients with suspected lower respiratory tract infections who underwent bronchoscopy and BALF collection were initially screened between January 2021 and December 2023. After excluding 87 patients for insufficient sample volume, incomplete clinical data, or withdrawal of informed consent, 941 patients were finally included in the analysis, with 479 assigned to the mNGS cohort and 462 to the tNGS cohort.

### 2.2 Specimen collection, conventional testing, and sequencing procedures

Flexible bronchoscopy was performed on all enrolled patients following standard clinical protocols. Sterile saline was instilled into the radiographically affected lung segments, and bronchoalveolar lavage fluid (BALF) specimens were collected. Aliquots of the BALF samples were immediately maintained on cold chain transport and sent to a central commercial laboratory (Beijing Dian Diagnostics, China) for high-throughput sequencing. Concurrently, routine conventional microbiological tests (CMTs) were performed at our institution, which included standard bacterial and fungal cultures, acid-fast bacilli (AFB) smears, and relevant molecular assays.

For patients allocated to the mNGS cohort, DNA-based metagenomic sequencing was performed. Total DNA was extracted from the BALF samples [using the standard extraction kit]. Sequencing libraries were constructed via DNA fragmentation, end-repair, and adapter ligation. Sequencing was executed on a high-throughput platform [Illumina]. Analyzing the mNGS data required rigorous bioinformatics processing. First, low-quality reads and short fragments were filtered out. Subsequently, human host sequences were subtracted by mapping the reads to the human reference genome. Finally, the remaining non-host reads were aligned against a comprehensive microbial database to identify putative pathogens.

For the tNGS cohort, testing utilized a multiplex PCR-based targeted enrichment panel encompassing over 500 common respiratory microbes, including bacteria, fungi, viruses, and atypical pathogens. Total nucleic acids were extracted, and RNA was reverse-transcribed into cDNA. Target enrichment was achieved through multiplex PCR amplification. The resulting amplicons were utilized for library preparation and subsequently sequenced. To distinguish clinically significant pathogens from colonizers and background contaminants, stringent bioinformatic read cutoff thresholds were applied in accordance with the laboratory’s standard diagnostic criteria.

### 2.3 Statistical analysis

All statistical analyses were performed using R software (version 4.2). Categorical variables were presented as frequencies and percentages, and group comparisons were conducted using the Chi-square test or Fisher’s exact test, as appropriate. Continuous variables were expressed as medians with interquartile ranges (IQRs) and evaluated using the Mann-Whitney U test. Diagnostic performance metrics, including sensitivity, specificity, positive predictive value (PPV), and negative predictive value (NPV), were calculated utilizing the final clinical diagnosis as the reference standard. The 95% confidence intervals (CIs) for all applicable diagnostic metrics were computed to estimate precision. A two-sided P-value of < 0.05 was considered statistically significant.

## 3 Results

### 3.1 Baseline characteristics, pathogen distribution, and mixed infections

The baseline demographic and clinical characteristics of the 941 enrolled patients are summarized in **Table 1**. Across the study cohort, both the tNGS and mNGS platforms demonstrated robust diagnostic capabilities, successfully identifying a diverse spectrum of microorganisms encompassing bacteria, viruses, and fungi. The comprehensive distribution of these detected pathogens is illustrated in **Figure 2**. Among the bacterial isolates, Klebsiella pneumoniae and Streptococcus pneumoniae were the most frequently detected etiologies. Regarding viral infections, Influenza A virus was the most predominant. Notably, an exceptionally high prevalence of mixed (polymicrobial) infections was observed in the study, particularly within the tNGS cohort (93.5%). The most frequent co-infection profiles involved a cross-kingdom combination of Streptococcus pneumoniae and Human herpesvirus 7 (HHV-7) (n = 30), followed closely by bacterial co-detections comprising Klebsiella pneumoniae and Streptococcus pneumoniae (n = 25). Furthermore, co-detections involving bacterial agents and opportunistic fungi were also highly prevalent, predominantly featuring Pneumocystis jirovecii alongside Pseudomonas aeruginosa (n = 17) or Klebsiella pneumoniae (n = 16).

**Table 1:** Baseline demographic and clinical characteristics of the study cohort.

Characteristics	Total (N = 941)	mNGS Cohort (n = 479)	tNGS Cohort (n = 462)	*P*-value
Demographics				
Age, median (IQR), years	61.0 (51.0–69.0)	61.0 (51.0–69.0)	61.0 (52.0–68.0)	0.842
Male gender, n (%)	540 (57.4%)	275 (57.4%)	265 (57.4%)	0.739
Underlying diseases & risk factors, n (%)				
Hypertension	113 (12.0%)	58 (12.1%)	55 (11.9%)	0.605
Diabetes mellitus	54 (5.7%)	28 (5.8%)	26 (5.6%)	0.658
Smoking history	44 (4.7%)	22 (4.6%)	22 (4.8%)	0.628
Clinical presentations, n (%)				
Cough	655 (69.6%)	333 (69.5%)	322 (69.7%)	0.617
Fever	310 (32.9%)	158 (33.0%)	152 (32.9%)	0.693
Dyspnea or wheezing	218 (23.2%)	111 (23.2%)	107 (23.2%)	0.640

Data are presented as median (interquartile range, IQR) for continuous variables and frequency (percentage) for categorical variables. Statistical comparisons between the mNGS and tNGS cohorts were performed using the Mann-Whitney U test for continuous variables and the Chi-square test for categorical variables. mNGS, metagenomic next-generation sequencing; tNGS, targeted next-generation sequencing.

**Figure 2:**
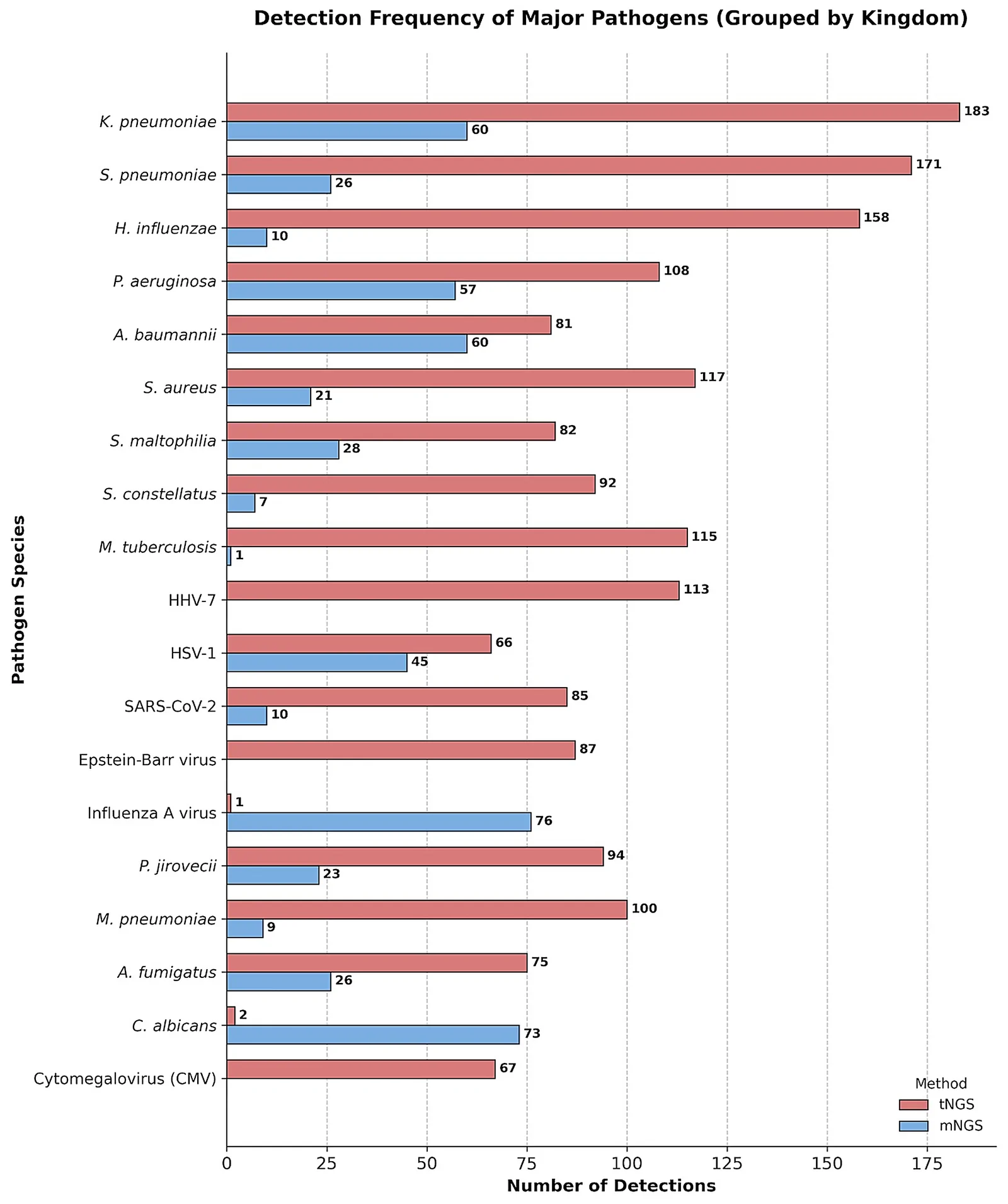
Detection frequency of major putative pathogens stratified by microbial kingdom. Pathogens detected by tNGS (red) and mNGS (blue) were categorized into bacteria, viruses, and fungi/atypical pathogens. The figure displays the most prevalent species within each category, providing a head-to-head comparison of diagnostic yield between the two sequencing platforms.

We found that single-pathogen infections were very rare. They accounted for only 6.5% (n = 22) of the tNGS-positive cohort. On the other hand, mixed infections were very common. We observed polymicrobial infections in 93.5% (n = 319) of the cases. The most frequent co-infection pattern was a mix of bacteria, fungi, and viruses. This cross-kingdom detection made up 32.6% (n = 111) of the positive cases.

### 3.2 Diagnostic performance and added yield

Utilizing the composite final clinical diagnosis as the reference standard, we evaluated the diagnostic performance of both sequencing platforms (**Figure 3**). Owing to its untargeted, pan-pathogen detection capability, mNGS exhibited a higher overall sensitivity of 90.6% (95% CI: 87.5%–93.0%) compared to 73.4% (95% CI: 69.2%–77.3%) for tNGS. Conversely, both methodologies demonstrated relatively low specificity, yielding 21.4% (95% CI: 7.6%–47.6%) for mNGS and 14.3% (95% CI: 4.0%–39.9%) for tNGS. This diminished specificity is frequently encountered in the high-throughput molecular testing of respiratory specimens, primarily due to the ubiquitous presence of commensal flora and airway colonizers that contribute to background genomic noise. Nevertheless, the positive predictive value (PPV) remained exceptionally robust for both assays—97.4% (95% CI: 95.3%–98.5%) for mNGS and 96.5% (95% CI: 94.0%–98.0%) for tNGS. These metrics indicate that positive pathogen identifications from either platform are highly reliable for guiding clinical interventions.

**Figure 3:**
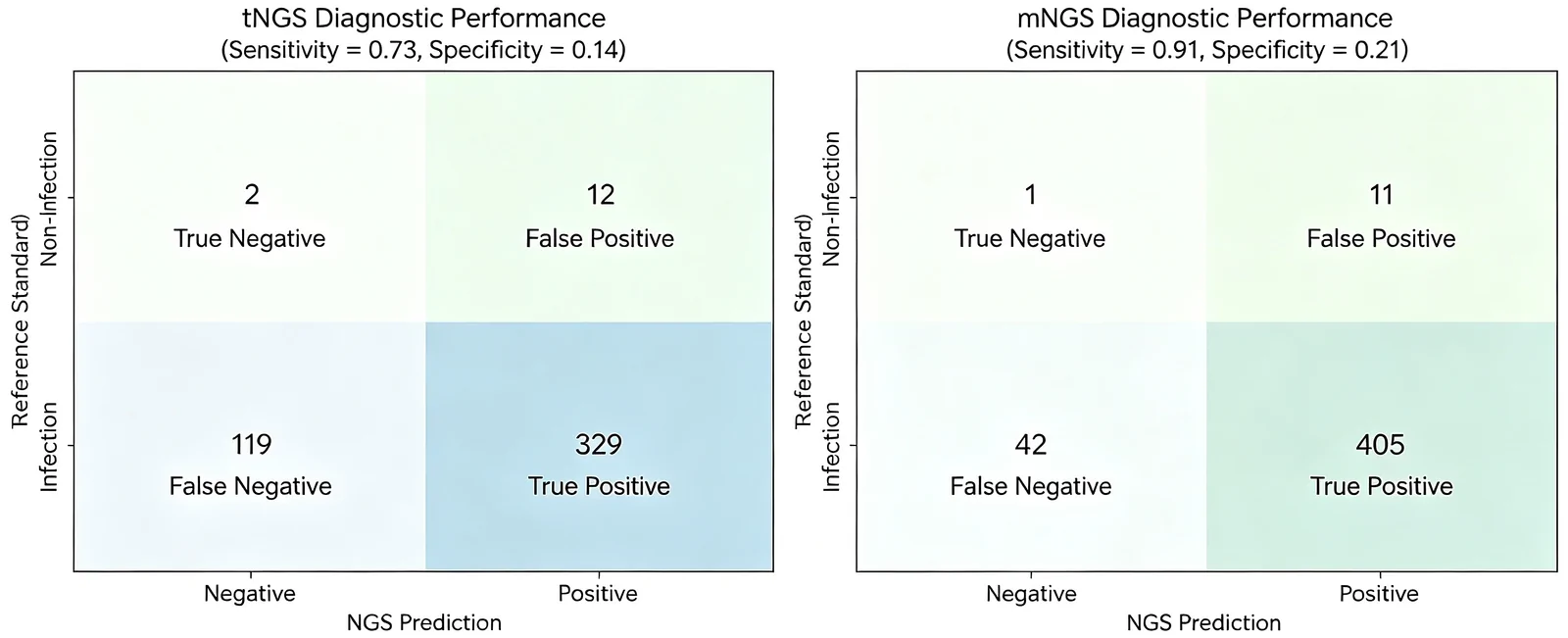
Diagnostic performance of sequencing platforms. Confusion matrices displaying the true positive, false positive, true negative, and false negative rates for tNGS and mNGS evaluated against the composite final clinical diagnosis reference standard.

Beyond fundamental diagnostic accuracy, both sequencing platforms demonstrated superior diagnostic yield compared to conventional microbiological tests (CMTs) in identifying elusive pathogens. Particularly, tNGS exhibited a distinct advantage in detecting fastidious organisms. Within the cohort, tNGS identified 54 cases of Mycobacterium tuberculosis infection; concurrently, traditional acid-fast bacilli (AFB) smears and routine nucleic acid amplification tests (NAATs) missed 42 of these positive cases, equating to a substantial false-negative rate of 77.8% for conventional diagnostic modalities. This augmented diagnostic value was equally evident in the detection of opportunistic pathogens such as Pneumocystis jirovecii. Among the cases evaluated, conventional silver staining yielded false-negative results in 80.0% (8/10) of instances, whereas tNGS successfully and rapidly established the precise microbiological diagnosis, thereby overcoming the sensitivity limitations inherent in traditional laboratory techniques.

### 3.3 Antibiotic management and clinical outcomes guided by NGS

The integration of high-throughput sequencing results significantly influenced clinical decision-making, particularly regarding antimicrobial stewardship. Within the tNGS cohort, a total of 341 patients yielded positive sequencing results. Guided by this targeted genomic data, attending physicians modified the initial empirical antimicrobial regimens in 23.2% of these cases (**Figure 4**). Specifically, treatment escalation was necessitated for 77 patients (22.6%), as their initial empirical therapies were retrospectively determined to provide inadequate coverage for the specific pathogens identified by tNGS.

**Figure 4:**
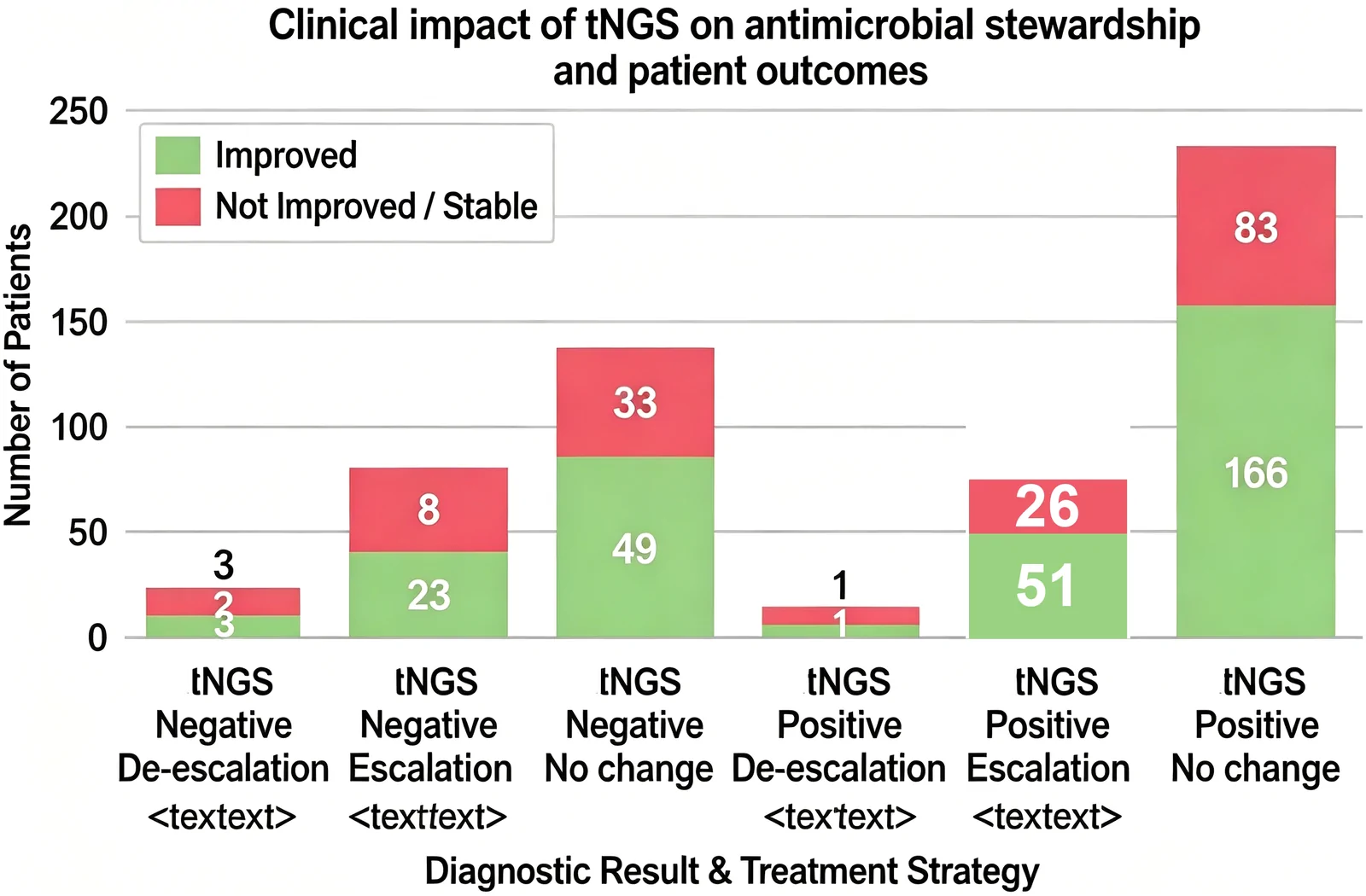
Clinical impact of tNGS on antimicrobial stewardship and patient outcomes. The stacked bar chart illustrates the proportion of patients achieving clinical and radiological improvement, stratified by treatment adjustment strategies (escalation, de-escalation, and no change) following tNGS reporting.

To ensure a comprehensive evaluation across both platforms, corresponding antimicrobial adjustments were also analyzed in the mNGS cohort. Among the 461 patients with positive mNGS results, initial empirical therapies were modified in 50.8% (n = 234) of cases based on the sequencing outputs, with targeted treatment escalation—such as the addition of broad-spectrum carbapenems, glycopeptides, or targeted antifungals—occurring in 184 patients (39.9%).

Following these antimicrobial adjustments, we evaluated the composite clinical and radiological outcomes. Among the 77 patients in the tNGS cohort who required treatment escalation, the clinical improvement rate reached 66.2% (51/77) after the targeted modification of their regimens. This was evaluated against the “no-change” cohort—comprising 249 patients whose initial empirical therapy was retrospectively confirmed to be appropriate by the genomic data, thereby prompting maintenance of the original regimen. The improvement rate in this cohort was 66.7% (166/249). Statistical analysis revealed no significant difference in the clinical recovery rates between the two groups (P = 0.946). These findings indicate that tNGS-guided therapy escalation effectively rescued potentially failing empirical regimens, allowing patients to achieve clinical outcomes comparable to those who received appropriate therapy from the outset.

## 4 Discussion

In this large-scale retrospective study encompassing 941 patients, we present one of the most comprehensive real-world comparative evaluations of tNGS and mNGS for diagnosing LRTIs. The diagnostic utility of BALF is frequently compromised by a substantial human cellular background, predominantly comprising leukocytes and airway epithelial cells. This high proportion of host DNA generates a well-documented “signal dilution” effect during sequencing. Consequently, in untargeted mNGS assays, a significant fraction of sequencing reads is inadvertently allocated to human genomic material, which can obscure the signals of genuine, low-abundance pathogens—a challenge similarly highlighted by Shi et al. (2024).

While our study did not directly quantify host-read fractions, sequencing depth allocation, or mapping statistics, our clinical findings indirectly support the premise that tNGS effectively mitigates this host DNA interference. By employing multiplex PCR-based targeted enrichment prior to library construction, tNGS selectively amplifies specific microbial sequences. This mechanism theoretically acts as a molecular filter, substantially reducing the human genomic background, which aligns with recent observations by Hu et al. (2024) and Zhang et al. (2024). The practical implication of this targeted approach is particularly evident in the detection of low-titer or fastidious organisms, such as Mycobacterium tuberculosis and Pneumocystis jirovecii. Traditional mycobacterial cultures are notoriously protracted, and conventional smears frequently yield false-negative results. Similarly, routine silver staining for P. jirovecii exhibits suboptimal sensitivity and often fails to detect the pathogen, a diagnostic limitation well-documented in recent literature (Zhou et al., 2024). In our cohort, tNGS rapidly and successfully identified these elusive pathogens, thereby overcoming the inherent sensitivity thresholds of conventional laboratory diagnostics (Wang et al., 2024).

A related challenge in the metagenomic testing of complex clinical specimens is the “read-depth allocation dilemma” (Sun et al., 2022). In BALF samples characterized by high human cellularity, untargeted mNGS may fail to allocate sufficient sequencing depth to sparse microbial targets. The targeted amplification step inherent in tNGS theoretically circumvents this limitation by concentrating sequencing reads specifically on microbial genomes. Furthermore, our study revealed an exceptionally high prevalence of mixed infections (93.5% in the tNGS cohort). Traditional CMTs, which often target single pathogens or require highly specific culture conditions, frequently fail to capture the full spectrum of polymicrobial infections. By providing a comprehensive profile of cross-kingdom co-infections (involving bacteria, viruses, and fungi simultaneously), tNGS offers a more complete etiological landscape.

Ultimately, the primary objective of advanced molecular diagnostics extends beyond mere pathogen identification to optimizing antimicrobial stewardship and improving patient outcomes (Wang et al., 2023). Severe LRTI patients typically receive broad-spectrum empirical antibiotics upon admission, which may prove inadequate against unexpected, fastidious, or resistant pathogens. In our cohort, targeted genomic data provided actionable insights that guided the escalation or modification of these initially failing regimens. Following these targeted adjustments, patients demonstrated favorable clinical trajectories.

However, these outcome data must be interpreted with caution. Patient recovery is multifactorial and heavily influenced by baseline disease severity, underlying comorbidities, and the quality of supportive care. Because our retrospective design precluded a robust multivariate logistic regression analysis to adjust for these confounders, the observed clinical improvements cannot be definitively established as the independent, direct result of NGS-guided therapy. Moreover, the routine implementation of tNGS in daily clinical practice must be carefully balanced against practical considerations. While offering superior diagnostic yield, NGS platforms currently demand higher testing costs, specialized bioinformatic infrastructure, and occasionally longer turnaround times compared to point-of-care rapid antigen tests or basic smears.

### 4.1 Limitations

Our study has several notable limitations that must be acknowledged. First, as a single-center retrospective study, our findings are inherently subject to selection bias and lack external validation. Second, the study lacked a paired-testing design. Patients were allocated to either the mNGS or tNGS cohort in a non-randomized, unblinded manner based on clinical and practical considerations. This precludes a direct, intra-patient head-to-head comparison of diagnostic superiority. Third, the definition of our reference standard—the final clinical diagnosis—may have been subject to incorporation bias, as the sequencing results were inevitably available to the treating clinicians and the diagnostic committee.

Fourth, as previously discussed, the retrospective nature of data collection resulted in missing baseline variables, such as comprehensive severity indices (e.g., APACHE II or SOFA scores) and detailed outpatient antibiotic exposure histories. This limitation prevented the execution of robust multivariate logistic regression analyses, restricting our clinical outcome findings to observational associations rather than independent causal effects. Fifth, a notable technical discrepancy existed between the platforms: the mNGS assay utilized in this study was restricted to DNA sequencing, whereas the tNGS panel enriched for both DNA and RNA. This inherent limitation likely underestimated the true prevalence of RNA viral infections within the mNGS cohort. Sixth, we did not quantitatively capture key sequencing metrics, such as exact host-to-microbe read ratios or sequencing depth allocation, limiting our ability to mechanically prove the extent of host DNA depletion. Finally, our evaluation was restricted to short-term clinical improvements. Future prospective, multicenter studies incorporating paired-testing and long-term follow-up are essential to determine the ultimate impact of sequencing diagnostics on overall patient survival rates.

## 5 Conclusion

In conclusion, this large-scale retrospective analysis of 941 patients underscores the substantial clinical utility of both mNGS and tNGS in the diagnostic workflow for lower respiratory tract infections (LRTIs). Both high-throughput sequencing platforms demonstrate significant diagnostic superiority over conventional microbiological tests, particularly in overcoming inherent limitations related to low sensitivity and prolonged turnaround times.

While the diagnostic yield of BALF samples is frequently hindered by a high human genomic background, the targeted enrichment strategy employed by tNGS appears to effectively mitigate this host DNA interference. Consequently, tNGS exhibits exceptional sensitivity for detecting low-titer and fastidious pathogens, successfully identifying critical cases of Mycobacterium tuberculosis and Pneumocystis jirovecii that are frequently missed by routine laboratory methods.

Beyond accurate pathogen detection, the rapid genomic data provided by these sequencing platforms plays a pivotal role in informing antimicrobial stewardship. By identifying precise etiological targets, NGS empowers clinicians to timely adjust failing empirical therapies—interventions that were closely associated with favorable clinical trajectories in our cohort. Although future prospective, paired-testing studies are warranted to definitively isolate the independent impact of sequencing on survival outcomes, our real-world findings affirm that tNGS is a highly reliable and practical diagnostic tool, effectively driving the clinical management of severe respiratory infections toward true precision medicine.

## Data Availability

The original contributions presented in the study are included in the article/supplementary material. Further inquiries can be directed to the corresponding author.
